# Effects of Benzodiazepine Exposure on Real-World Clinical Outcomes in Individuals at Clinical High Risk for Psychosis

**DOI:** 10.1093/schbul/sbae036

**Published:** 2024-04-03

**Authors:** Nicholas R Livingston, Andrea De Micheli, Robert A McCutcheon, Emma Butler, Marwa Hamdan, Anthony A Grace, Philip McGuire, Alice Egerton, Paolo Fusar-Poli, Gemma Modinos

**Affiliations:** Department of Psychological Medicine, Institute of Psychiatry, Psychology & Neuroscience, King’s College London, London, UK; Early Psychosis: Interventions & Clinical-detection (EPIC) Lab, Department of Psychosis Studies, Institute of Psychiatry, Psychology & Neuroscience, King’s College London, London, UK; Outreach And Support in South London (OASIS) Service, South London and Maudsley NHS Foundation Trust, London, UK; Department of Psychiatry, University of Oxford, Oxford, UK; Oxford Health NHS Foundation Trust, Oxford, UK; Department of Psychosis Studies, Institute of Psychiatry, Psychology & Neuroscience, King’s College London, London, UK; Department of Psychosis Studies, Institute of Psychiatry, Psychology & Neuroscience, King’s College London, London, UK; Department of Psychosis Studies, Institute of Psychiatry, Psychology & Neuroscience, King’s College London, London, UK; Department of Neuroscience, University of Pittsburgh, Pittsburgh, PA, USA; Department of Psychiatry, University of Pittsburgh, Pittsburgh, PA, USA; Department of Psychology, University of Pittsburgh, Pittsburgh, PA, USA; Department of Psychiatry, University of Oxford, Oxford, UK; Oxford Health NHS Foundation Trust, Oxford, UK; National Institute for Health Research (NIHR) Oxford Health Biomedical Research Centre (BRC), Oxford, UK; Department of Psychosis Studies, Institute of Psychiatry, Psychology & Neuroscience, King’s College London, London, UK; National Institute of Health Research (NIHR), Maudsley Biomedical Research Centre (BRC), South London and Maudsley NHS Foundation Trust, London, UK; Early Psychosis: Interventions & Clinical-detection (EPIC) Lab, Department of Psychosis Studies, Institute of Psychiatry, Psychology & Neuroscience, King’s College London, London, UK; Outreach And Support in South London (OASIS) Service, South London and Maudsley NHS Foundation Trust, London, UK; Department of Brain and Behavioural Sciences, University of Pavia, Pavia, Italy; National Institute of Health Research (NIHR), Maudsley Biomedical Research Centre (BRC), South London and Maudsley NHS Foundation Trust, London, UK; Department of Psychological Medicine, Institute of Psychiatry, Psychology & Neuroscience, King’s College London, London, UK; MRC Centre for Neurodevelopmental Disorders, King’s College London, London, UK

**Keywords:** GABA, health records, prevention, propensity score matching, survival analysis

## Abstract

**Background and Hypothesis:**

Animal models indicate GABAergic dysfunction in the development of psychosis, and that benzodiazepine (BDZ) exposure can prevent the emergence of psychosis-relevant phenotypes. However, whether BDZ exposure influences real-world clinical outcomes in individuals at clinical high risk for psychosis (CHR-P) is unknown.

**Study Design:**

This observational cohort study used electronic health record data from CHR-P individuals to investigate whether BDZ exposure (including hypnotics, eg, zopiclone) reduces the risk of developing psychosis and adverse clinical outcomes. Cox proportional-hazards models were employed in both the whole-unmatched sample, and a propensity score matched (PSM) subsample.

**Study Results:**

567 CHR-P individuals (306 male, mean[±SD] age = 22.3[±4.9] years) were included after data cleaning. The BDZ-exposed (*n* = 105) and BDZ-unexposed (*n* = 462) groups differed on several demographic and clinical characteristics, including psychotic symptom severity. In the whole-unmatched sample, BDZ exposure was associated with increased risk of transition to psychosis (HR = 1.61; 95% CI: 1.03–2.52; *P* = .037), psychiatric hospital admission (HR = 1.93; 95% CI: 1.13–3.29; *P* = .017), home visit (HR = 1.64; 95% CI: 1.18–2.28; *P* = .004), and Accident and Emergency department attendance (HR = 1.88; 95% CI: 1.31–2.72; *P* < .001). However, after controlling for confounding-by-indication through PSM, BDZ exposure did not modulate the risk of any outcomes (all *P* > .05). In an analysis restricted to antipsychotic-naïve individuals, BDZ exposure *reduced* the risk of transition to psychosis numerically, although this was not statistically significant (HR = 0.59; 95% CI: 0.32–1.08; *P* = .089).

**Conclusions:**

BDZ exposure in CHR-P individuals was not associated with a reduction in the risk of psychosis transition or adverse clinical outcomes. Results in the whole-unmatched sample suggest BDZ prescription may be more likely in CHR-P individuals with higher symptom severity.

## Introduction

Psychotic disorders are among the most severe psychiatric disorders, associated with chronic functional disability,^1^ poor physical health, reduced life expectancy,^2^ and high personal and familial burden.^3^ Efforts to prevent the onset of psychosis in those at clinical high risk for psychosis (CHR-P) have so far been unsuccessful.^4,5^ CHR-P individuals display attenuated psychotic symptoms (APS) or a first-degree familial risk, and about 25% of them will develop psychosis within 3 years.^6,7^ Almost 50% of those who do not develop psychosis remain in the CHR-P state,^8^ associated with reduced functioning and quality of life,^9^ neurocognitive impairments,^10,11^ and increased mental health resource utilization, including Accident and Emergency department (A&E) attendance and psychiatric hospital admission.^12,13^ However, there is no evidence to favor any available pharmacological interventions for reducing transition to psychosis,^14,15^ and more recent multicenter clinical trials of cognitive behavioral therapy^16^ have failed to replicate positive findings.^15,17^ There is thus a substantial need to develop interventions to prevent the onset of psychosis and improve clinical outcomes in CHR-P individuals.

Psychotic symptoms are associated with increased dopamine release in the striatum,^18^ which is likely to occur from upstream pathophysiological mechanisms.^19^ A key mechanism is dysfunction of the gamma-aminobutyric acid (GABA) system in the hippocampus,^20^ leading to increased glutamatergic drive to the striatum, and consequently increased striatal dopaminergic neuron firing and dopamine release.^21^ This is supported by recent in vivo longitudinal neuroimaging evidence in antipsychotic-naïve individuals with a first episode of psychosis (FEP), showing that baseline prefrontal GABA levels—assessed with magnetic resonance spectroscopy—were reduced compared to healthy controls and negatively associated with striatal cerebral blood flow.^22^ Furthermore, the association between prefrontal GABA levels and striatal activity—assessed with^18^ F-DOPA position emission tomography—was able to distinguish FEP patients from healthy controls with greater accuracy than each neuroimaging measure separately.^23^ This evidence supports the notion that GABAergic and dopaminergic abnormalities are interrelated in early psychosis.^21^

Preclinical work in a well-validated rodent neurodevelopmental model—involving embryonic administration of methylazoxymethanol acetate (MAM)—with relevance to psychosis has demonstrated that peripubertal repeated administration of the GABA-enhancing drug diazepam, a benzodiazepine (BDZ), can prevent the emergence of both dopamine system hyperresponsivity and psychosis-relevant behavioral phenotypes at adulthood.^24–26^ In antipsychotic-free patients with schizophrenia, diazepam (vs placebo) has been demonstrated to be efficacious in preventing symptom progression.^27^ Additionally, the therapeutic effect of a more specific compound targeting α5-GABA_A_ receptor subunits in MAM-treated rats is blocked by previous exposure to the antipsychotic drug haloperidol.^28^ These findings suggest that enhancing GABAergic signaling during the premorbid phase of psychosis, prior to antipsychotic treatment, may prevent the development of psychosis. Whether the therapeutic potential of BDZs at the preclinical level translates to humans in early adulthood remains to be investigated.

Based on the above preclinical findings,^24,25,28,29^ we conducted a naturalistic, retrospective, observational cohort-design study using electronic health record (EHR) data from a large sample of CHR-P individuals to investigate the effect of BDZ exposure on clinical outcomes. We hypothesized that BDZ exposure would reduce the risk of transition to psychosis and events indicative of a clinical crisis: psychiatric hospital admission, home visit, and A&E attendance. Due to preclinical evidence suggesting a masking effect by prior antipsychotic treatment,^28^ a sensitivity analysis was also performed excluding CHR-P individuals with prior antipsychotic exposure.

## Methods

The authors assert that all procedures contributing to this work comply with the ethical standards of the relevant national and institutional committees on human experimentation and with the Helsinki Declaration of 1975, as revised in 2008. All procedures were approved by South London and Maudsley NHS Foundation Trust Psychosis clinical academic group.

### Study Design, Setting, and Population

**Fig. 1.**
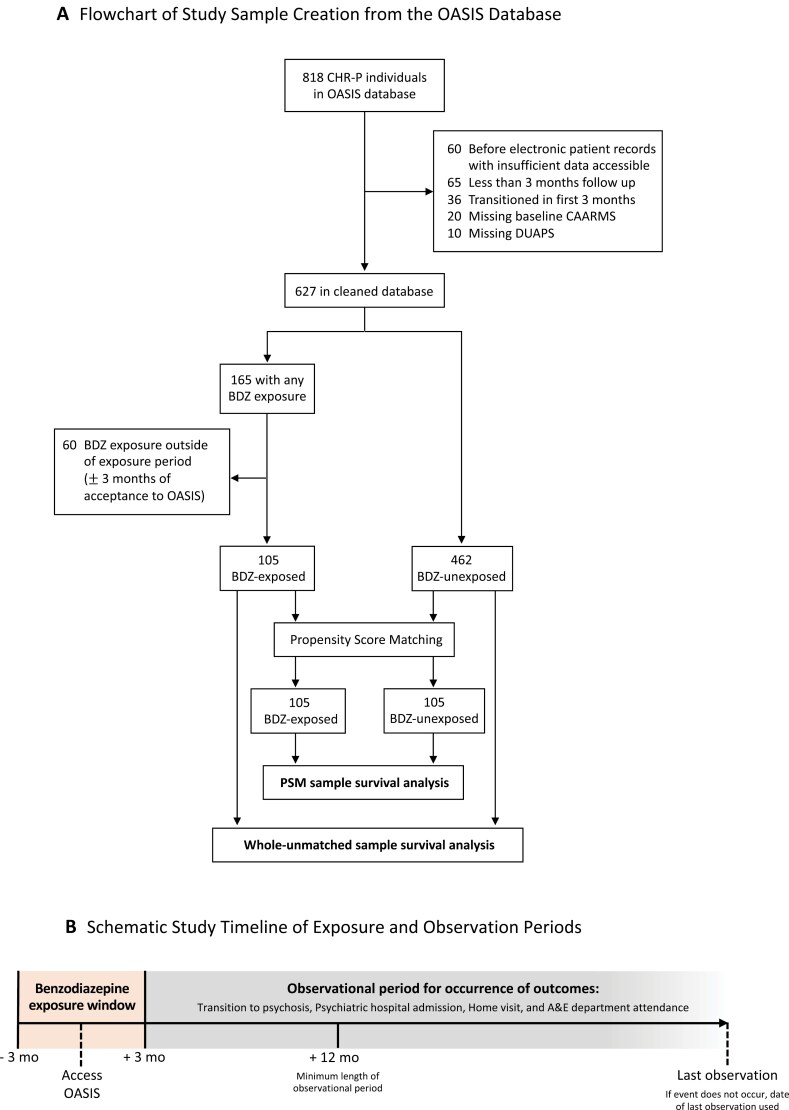
Study design. Flowchart of study sample creation from the OASIS database (A) and schematic study timeline of exposure and observation periods (B). Reasons for exclusion of ineligible participants from the study and survival analyses completed in the whole-unmatched sample and the subset sample created through propensity score matching. Benzodiazepine exposure window was operationalized as ±3 months of time joining OASIS. Observational periods begun at 3 months after accessing OASIS, and minimum follow-up period was operationalized at 9 months after this. Time-to-event was used for primary and exploratory secondary outcomes, and when an event did not occur date of last observation was used. BDZ, benzodiazepine; CAARMS, Comprehensive Assessment of At-Risk Mental State; CHR-P, clinical high risk for psychosis; DUAPS, duration of untreated attenuated psychotic symptoms; OASIS, Outreach And Support In South London; PSM, propensity score matching.

This observational cohort study used EHR data from CHR-P individuals accessing OASIS (Outreach And Support In South London),^30^ a CHR-P service within the South London and Maudsley National Health Service Foundation Trust, London, United Kingdom.^31^ CHR-P individuals are at an enhanced risk of developing psychosis due to either subthreshold psychotic symptoms in intensity (APS) or frequency (brief limited intermitted psychotic symptoms [BLIPS]), or by having a first-degree relative with a psychotic disorder and a recent decline in functioning (genetic risk and deterioration [GRD]).^4^ Data collection was between 2001 and 2021, and final data analysis was completed September–December 2022. To allow for a minimum follow-up period of 12 months, individuals who joined OASIS after September 2021 were excluded. Details regarding data cleaning and a schematic timeline of the exposure/observation periods are presented in figure 1.

### BDZ Exposure

BDZ exposure was operationalized as ≥1 dose of a BDZ within the exposure window (3 months prior to 3 months after accessing OASIS), and total number of days of BDZ exposure was calculated across the exposure window. BDZ exposure included non-BDZ hypnotics, eg, zopiclone, due to their very similar pharmacological mechanism of action via full agonism of the BDZ binding site on GABA_A_ receptors.^32^ BDZ-unexposed individuals were defined as having no previously recorded exposure to BDZs, according to EHR data, in the time prior to accessing OASIS up until the end of their observation period. Each individual’s observation period was defined as 3 months after accessing OASIS to date of last event, or when an event did not occur, date of last observation. Individuals who received a BDZ outside the exposure window but within the observation period were removed from the analysis.

### Primary and Exploratory Secondary Outcomes

The primary outcome was diagnosis of an ICD-10 psychotic disorder (F19.15, F20, F22, F23, F25, F29, F31, F31.2, F32.3, F33.3, and F1x.5) within the observation period. Exploratory secondary outcomes were psychiatric hospital admission, home visit, and A&E attendance. A home visit is undertaken in instances when there is an indication of deterioration in the client’s mental state in the presence of risk.

### Statistical Analysis

Statistical analyses were carried out using R (version 4.2.2). All statistical tests were 2-sided and statistical difference was set at *P* < .05. Analyses relating to secondary outcomes and additional sensitivity analyses were not adjusted for multiple comparisons as these were exploratory.

#### Propensity Score Matching.

As this study used real-world observational data, clinical and demographic factors may influence both the likelihood of BDZ exposure and the study’s primary and secondary outcomes, resulting in confounding-by-indication. We have recently discussed this issue in a letter to the Editor^33^ regarding a new meta-analysis that found BDZ exposure was associated with an increased risk of transition to psychosis in CHR-P individuals.^34^ To address this, a comparator BDZ-unexposed group was created through propensity score matching (PSM) using the MatchIt^35^ package in R. PSM involves running a logistic regression model on the whole database, with the binary outcome variable indicating BDZ exposure within the exposure window. The following covariates were entered into the model, which have been previously associated with BDZ prescription and/or our clinical outcome variables of interest^12^: APS severity when joining OASIS (specifically the total score of “unusual thought content” and “non-bizarre ideas” subscales^36,37^ on the CAARMS^38^ [Comprehensive Assessment of At-Risk Mental States] instrument), age, black ethnicity, duration of untreated APS (DUAPS), date of joining OASIS, and the occurrence of BLIPS. The model estimates were then used to calculate a propensity score for each individual, which represents the predicted probability of being exposed to a BDZ given these covariates. Each BDZ-exposed individual was then matched using the nearest neighbor method with a BDZ-unexposed individual with a near identical propensity score, resulting in 2 equally sized matched groups. The success of the PSM was assessed as the similarity of covariates between the groups using chi-square and *t* tests for categorical and continuous variables, respectively.

#### Survival Analysis.

Cox proportional-hazards models investigated whether BDZ exposure modulated the risk of primary and exploratory secondary outcomes. Individual models were run for each outcome, in the whole-unmatched sample and in the PSM sample. The proportional hazard assumption was used to check that hazards remained constant over time for each outcome.

#### Sensitivity Analyses.

In a primary sensitivity analysis, individuals with antipsychotic exposure prior to their BDZ exposure were removed from the database before statistical analysis. Three supplementary sensitivity analyses were run: (1) ≥ 3 or (2) ≥ 7 total days of BDZ exposure, and (3) removing non-BDZ hypnotics (eg, zopiclone).

## Results

### Data Cleaning and Final Sample Characteristics

**Fig. 2.**
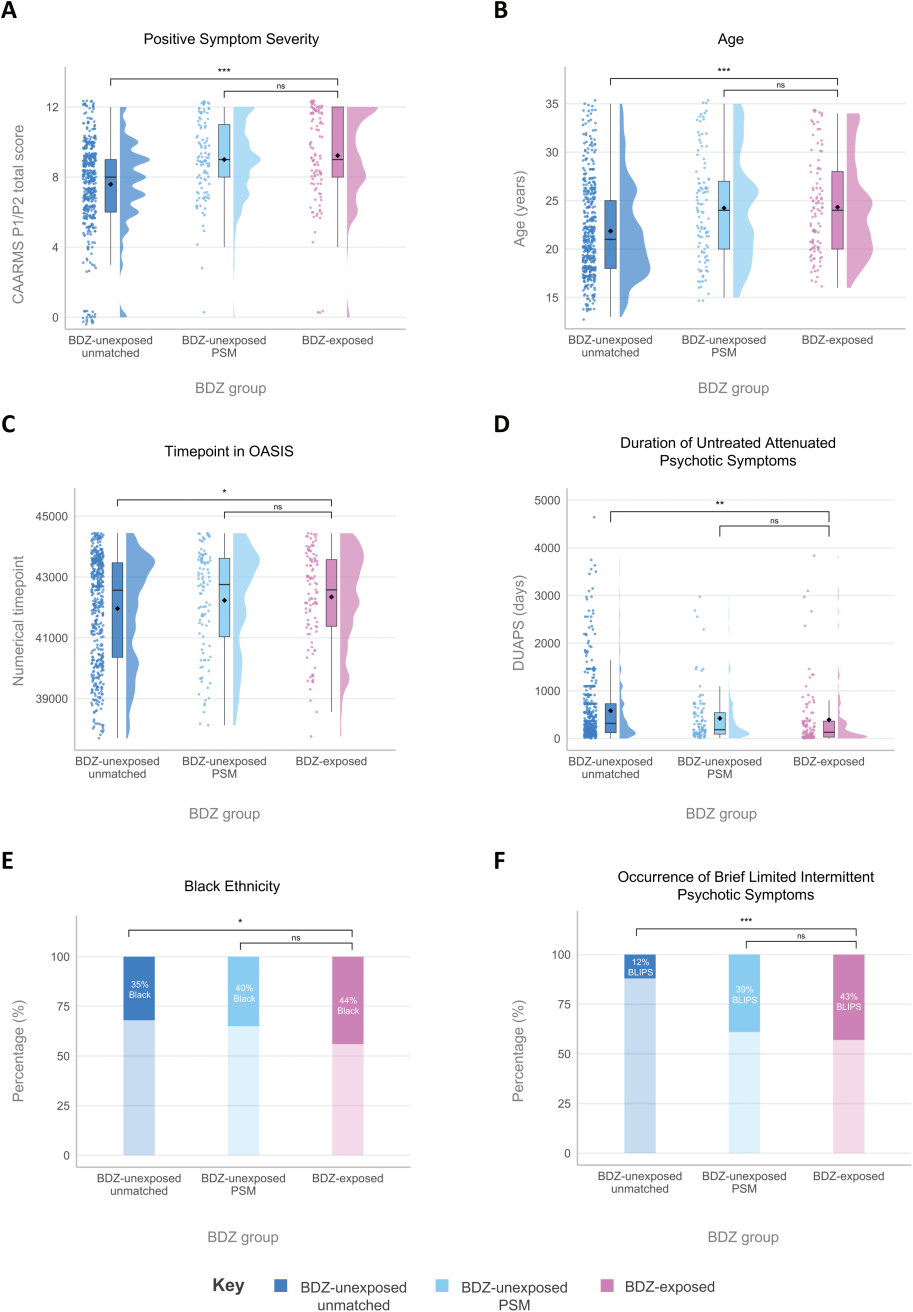
Demographic and clinical characteristics used to generate propensity scores for propensity score matching. Propensity score matching (PSM) successfully matched BDZ-exposed to BDZ-unexposed individuals on characteristics previously associated either with BDZ exposure or psychosis risk (A–F). On each panel, the left group (dark blue) represents BDZ-unexposed individuals in the whole sample, the middle group (light blue) represents a subset generated through PSM, and the right group (pink) represents BDZ-exposed individuals. *P < .05, **P < .01, ***P < .001, ns: nonsignificant. BDZ, benzodiazepine; BLIPS, brief limited intermittent psychotic symptoms; CAARMS, Comprehensive Assessment of At-Risk Mental State; DUAPS, duration of untreated attenuated psychotic symptoms; OASIS, Outreach And Support In South London; PSM, propensity score matching.

From the 818 CHR-P individuals in the OASIS database, 567 individuals were included (mean [SD] age 22.3 years [±4.9]; 306 [54%] male; 261 [46%] female) following data cleaning (figure 1; table 1). In the cleaned sample, 101 individuals transitioned to psychosis during their observation period. Most transitions occurred within the first 24 months after accessing OASIS (*n* = 85 <24 months; *n* = 16 ≥24 months). The sample included 105 individuals with BDZ exposure (mean [±SD] follow-up = 1157 [±1070] days) and 462 BDZ-unexposed individuals (mean [±SD] follow-up = 1190 [±1163] days). The median total number of days of BDZ exposure was 7 (IQR 3–21) days across the whole 6-month exposure window. The most common BDZ was zopiclone (51%), and the most common reason for prescription was for sleep (56%; supplementary table 1). Concomitant medication use in the BDZ-exposed group was relatively high for antipsychotics (55%) and antidepressants (51%; supplementary table 2). Within the final database of 567 individuals, compared to the BDZ-unexposed group, the BDZ-exposed individuals were more likely to be older (mean 24.3 vs 21.9 years), of black ethnicity (46 [44%] vs 148 [32%]), be classified within the BLIPS CHR-P subgroup (45 [43%] vs 55 [12%]), have higher psychotic symptom severity at baseline assessment (mean 9.2 vs 7.6 score out of 12), have a shorter DUAPS (mean 392 vs 338 days), and have accessed OASIS more recently (mean 42345 vs 41964 timepoint, made by converting date to numerical number). After PSM, there was no statistical difference between the groups on these variables, demonstrating the matching was successful (table 1; figure 2).

**Table 1.** Demographic and Clinical Characteristics of Individuals in the Whole-Unmatched and Propensity Score Matched Samples

Characteristic	BDZ-exposed (*n* = 105)	BDZ-unexposed unmatched (*n* = 462)	BDZ-exposed vs BDZ-unexposed whole-unmatched sample (*n* = 567)	BDZ-unexposed PSM (*n* = 105)	BDZ-exposed vs BDZ-unexposed PSM sample (*n* = 210)
	Mean (±*SD*)	Mean (±*SD*)	*t*/*χ*^2^ (*df*), *P*	Mean (±*SD*)	*t/χ* ^2^ (*df*), *P*
Age (years)	24.3 (4.9)	21.9 (4.9)	−4.63 (152.1), **<.001**	24.2 (5.4)	−0.12 (206.2), .91
CAARMS P1–P2 total	9.2 (2.7)	7.6 (2.6)	−5.65 (148.3), **<.001**	9.0 (2.3)	−0.66 (201.2), .51
DUAPS (days)	392 (676)	639 (891)	3.16 (195.5), **.002**	470 (737)	0.79 (206.4), 0.43
Timepoint in OASIS	42345 (1552)	41964 (1763)	−2.21 (170.6), **.028**	42231 (1731)	−0.50 (205.6), .62

	Count (%)	Count (%)	*t*/*χ*^2^ (*df*), *P*	Count (%)	*t*/*χ*^2^(*df*), *P*
Sex			4.4 × 10^−31^ (1), 1		0.08 (1), .78
Male	57 (54)	249 (53.8)	—	60 (57.1)	—
Female	48 (46)	213 (46.2)	—	45 (42.9)	—
Ethnicity					
White	45 (43)	213 (46.1)	0.28 (1), .59	40 (38.1)	0.26 (1), .61
Asian	4 (38)	42 (9.1)	2.56 (1), .11	12 (11.4)	3.39 (1), .068
Black^a^	46 (44)	146 (31.6)	5.16 (1), **.018**	37 (35.2)	1.23 (1), .26
Other	10 (10)	61 (13.2)	0.59 (1), .44	16 (15.2)	0.77 (1), .38
Type of CHR-P subgroup					
APS^b^	58 (55)	399 (86)	21.2 (1), **<.001**	64 (61)	0.17 (1), .68
GRD	2 (1.9)	6 (12.9)	2.8 × 10^−4^ (1), .98	0 (0.0)	0.50 (1), .48
BLIPS^c^	45 (42.9)	57 (12.3)	51.97 (1), **<.001**	41 (39.0)	0.18 (1), .67

^a^Black ethnicity includes Black African, Black Caribbean, and Black British.

^b^APS also includes APS + GRD.

^c^BLIPS also includes BLIPS + APS, BLIPS + GRD, and BLIPS + APS + GRD.

*Note:* APS, attenuated psychotic symptoms; BDZ, benzodiazepine; BLIPS, Brief Limited Intermittent Psychotic Symptoms; CAARMS, Comprehensive Assessment of At-Risk Mental State; CHR-P, clinical high risk for psychosis; DUAPS, duration of untreated attenuated psychotic symptoms; GRD, genetic risk and deterioration; OASIS, Outreach And Support In South London; PSM, propensity score matching.

### Effects of BDZ Exposure on Clinical Outcomes in the Whole-Unmatched Sample

**Fig. 3.**
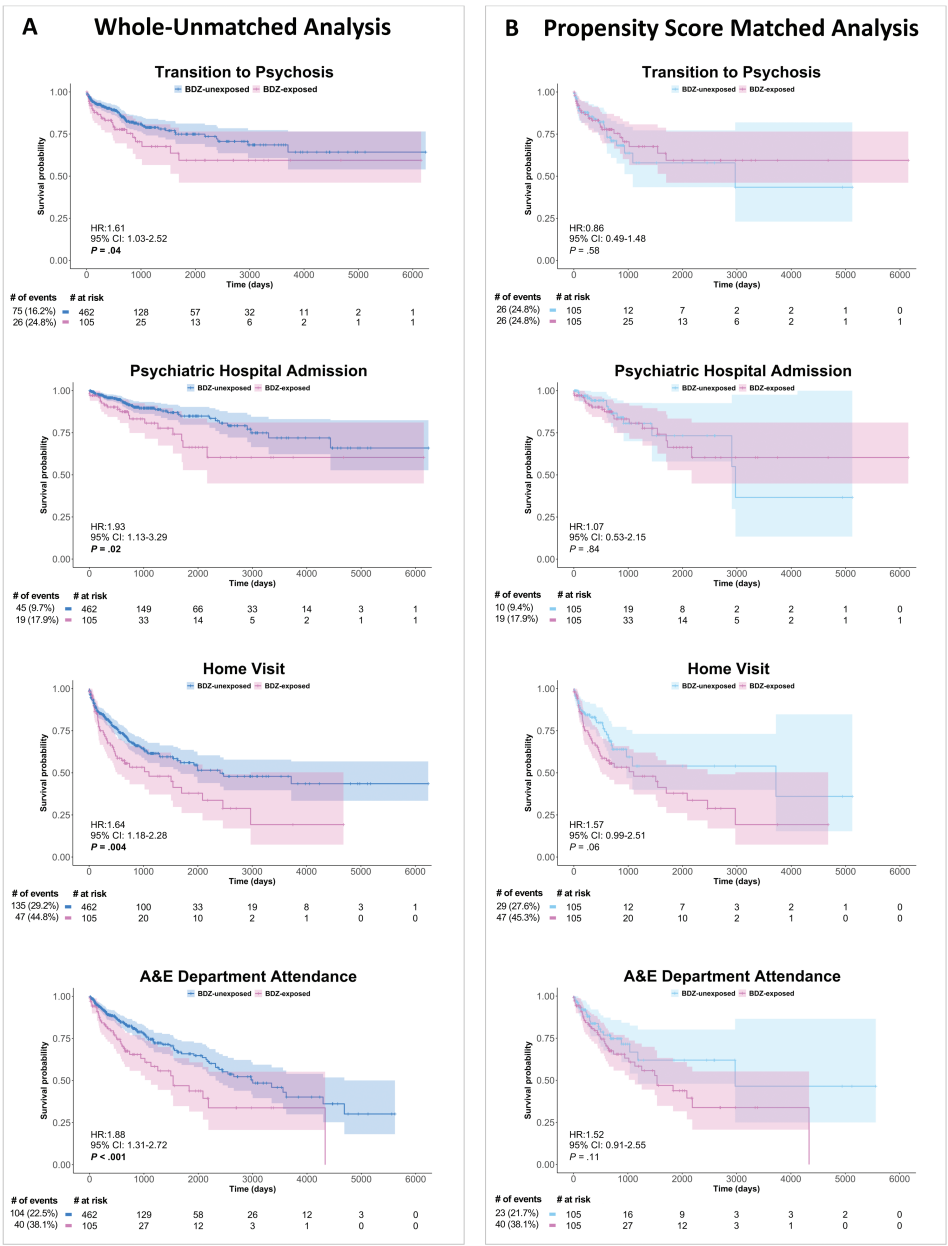
Survival analysis comparing the effect of benzodiazepine exposure on the risk of subsequent adverse clinical outcomes. Cox proportional-hazards models assessed the effects of BDZ exposure on the 4 outcome variables for the whole-unmatched sample (A) and PSM sample (B), displayed on Kaplan–Meier survival curves, with the Cox proportional-hazard ratios, confidence intervals, and *P* values displayed on each curve and a risk table beneath. N.B. Caution should be used when interpreting Kaplan–Meier curves when <10 individuals are at risk due to high levels of noise. The curves are used for display purposes only, and statistical analyses were only conducted on the cox proportional-hazards models. A&E, Accident and Emergency; BDZ, benzodiazepine; PSM, propensity score matching.

In the whole-unmatched sample (figure 3A), BDZ exposure was associated with an *increased* risk of transition to psychosis (HR = 1.61; 95% CI: 1.03–2.52; *P* = .037; No. [%] of events for BDZ-exposed vs BDZ-unexposed = 26 [24.8%] vs 75 [16.2%]). There was no significant interaction between BDZ exposure and sex on the risk of transition to psychosis (HR = 0.47; 95% CI: 0.19–1.16; *P* = .104). Similar results were found for the exploratory secondary outcomes, as BDZ exposure was associated with increased risk of psychiatric hospital admission (HR = 1.93; 95% CI: 1.13–3.29; *P* = .017; No. [%] of events BDZ-exposed vs BDZ-unexposed = 19 [17.9%] vs 45 [9.7%]), home visit (HR = 1.64; 95% CI: 1.18–2.28; *P* = .004; No. [%] of events BDZ-exposed vs BDZ-unexposed = 47 [44.8%] vs 135 [29.2%]), and A&E attendance (HR = 1.88; 95% CI: 1.31–2.72; *P* < .001; No. [%] of events BDZ-exposed vs BDZ-unexposed = 26 [24.8%] vs 75 [16.2%]). The proportionality assumption was met for all 4 models (*χ*^2^ = 0.54, *P* = .46; *χ*^2^ = 0.41, *P* = .52; *χ*^2^ = 0.49, *P* = .48; *χ*^2^ = 15, *P* = .69, respectively).

### Effects of BDZ Exposure on Clinical Outcomes in the PSM Sample

In the PSM sample (figure 3B), BDZ exposure did not modulate the risk of transition to psychosis (HR = 0.86; 95% CI: 0.49–1.48; *P* = .58; No. [%] of events BDZ-exposed vs BDZ-unexposed = 26 [24.8%] vs 26 [24.8%]). Similar results were found for the exploratory secondary outcomes, as BDZ exposure did not modulate the risk of a psychiatric hospital admission (HR = 1.07; 95% CI: 0.53–2.15; *P* = .84; No. [%] of events BDZ-exposed vs BDZ-unexposed = 19 [17.9%] vs 10 [9.4%]), home visit (HR = 1.57; 95% CI: 0.99–2.51; *P* = .055; No. [%] of events BDZ-exposed vs BDZ-unexposed = 47 [44.8%] vs 29 [27.6%]), or A&E attendance (HR = 1.52; 95% CI: 0.91–2.55; *P* = .11; No. [%] of events BDZ-exposed vs BDZ-unexposed = 40 [38.1%] vs 23 [21.7%]). The proportionality assumption was met for all 4 models (*χ*^2^ = 0.85, *P* = .36; *χ*^2^ = 1.46, *P* = .23; *χ*^2^ = 0.05, *P* = .83; *χ*^2^ = 1.01, *P* = .31, respectively).

### Sensitivity Analyses

**Fig. 4.**
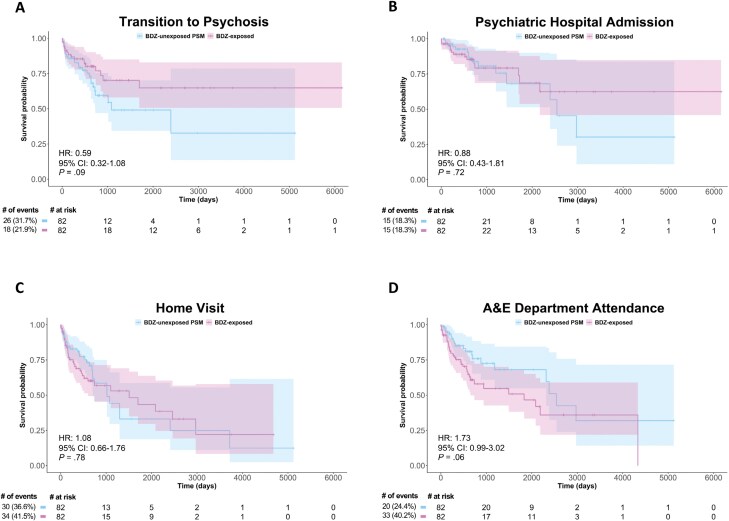
Sensitivity analysis removing individuals with prior antipsychotic exposure. Cox proportional-hazards models assessed the effect of BDZ exposure in antipsychotic-naïve individuals in the PSM sample on the 4 outcome variables (A–D), displayed on Kaplan–Meier survival curves, with the Cox proportional-hazard ratios, confidence intervals, and *P* values displayed on each curve and a risk table beneath. N.B. Caution should be used when interpreting Kaplan–Meier curves when <10 individuals are at risk due to high levels of noise. The curves are used here for display purposes only, and statistical analyses were only conducted on the cox proportional-hazards models. A&E, Accident and Emergency; BDZ, benzodiazepine; PSM, propensity score.

Removing individuals with prior antipsychotic exposure (*n* = 23) revealed that BDZ exposure (in *n* = 82) numerically reduced the risk of transition to psychosis, although this did not reach significance (HR = 0.59; 95% CI: 0.32–1.08; *P* = .089; No. [%] of events BDZ-exposed vs BDZ-unexposed = 23 [21.9%] vs 33 [31.7%]; figure 4A). BDZ exposure did not modulate the risk of psychiatric hospital admission (HR = 0.88; 95% CI: 0.43–1.81; *P* = .73; No. [%] of events BDZ-exposed vs BDZ-unexposed = 19 [18.3%] vs 19 [18.3%]; figure 4B), home visit (HR = 1.08; 95% CI: 0.66–1.76; *P* = .78; No. [%] of events BDZ-exposed vs BDZ-unexposed = 44 [41.5%] vs 38 [36.6%]; figure 4C), or A&E attendance (HR = 1.73; 95% CI: 0.99–3.02; *P* = .054; No. [%] of events BDZ-exposed vs BDZ-unexposed = 42 [40.2%] vs 26 [24.4%]; figure 4D). The proportionality assumption was met for all 4 models (*χ*^2^ = 1.49, *P* = .22; *χ*^2^ = 3.69, *P* = .063; *χ*^2^ = 1.22, *P* = .27; *χ*^2^ = 1.47, *P* = .22, respectively). Finally, supplemental sensitivity analyses showed no significant effects of BDZ exposures for 3 or 7 days on any clinical outcomes, and removing non-BDZ hypnotics from the analyses did not change the results, except for a significantly increased risk of A&E attendance (supplementary table 2).

## Discussion

To the best of our knowledge, this is the first study using EHR data to investigate the hypothesis that BDZ exposure can improve real-world clinical outcomes in a large sample of CHR-P individuals. In the whole-unmatched sample, BDZ exposure instead was associated with an *increased* risk of developing a psychotic disorder, psychiatric hospital admission, home visit, and A&E attendance. However, after employing propensity score matching (PSM) to account for confounding-by-indication, BDZ exposure did not modulate the risk of transition to psychosis or other events indicative of a clinical crisis. Restricting the analysis to individuals with no prior antipsychotic exposure suggested that BDZ exposure numerically reduced the risk of transition to psychosis, although this did not reach significance.

Following PSM, the increased risk of transition to psychosis associated with BDZ exposure in the whole-unmatched sample was removed. This suggests confounding-by-indication, such that BDZs are prescribed to individuals who are clinically more unwell, or have a demographic background associated with a higher risk for transition and are therefore already more likely to develop a psychotic disorder. This misleading association was recently observed in a meta-analysis, which reported that BDZ exposure was associated with an increased risk of transition to psychosis in CHR-P individuals^34^ as confounding-by-indication was not considered.^33^ While controlling for these confounds in the PSM analysis of this study removed the increased risk, it did not demonstrate a protective effect of BDZ exposure on psychosis risk as we had hypothesized. Interestingly, when we removed individuals with prior antipsychotic exposure, the hazard ratio for transition to psychosis dropped from 0.86 to 0.59, suggesting a protective effect of the BDZ exposure on psychosis risk in antipsychotic-naïve individuals. We conducted this sensitivity analysis based on preclinical findings that chronic haloperidol treatment blocks the subsequent effects of a selective GABA-enhancing compound (an α5GABA_A_R positive allosteric modulator [PAM]) in MAM-treated rats,^28^ likely due to postsynaptic D2-receptor supersensitivity. However, there was also a borderline significant increased risk of attending A&E in the antipsychotic-naïve BDZ-exposed group. Hence, the preventative effect of BDZs warrants further investigation due to its potential clinical significance and to ensure its effects are not overestimated.

The many differences in experimental model and exposure could explain the lack of convergence between preclinical findings and our observations in humans for the primary outcome of transition to psychosis. As with any animal model, the MAM model will have limited validity in capturing the complex neurobiology and outward expressed phenotype of psychosis. Differences in BDZ compound, dose equivalence, and length of dosing period may also be important. We ran several additional sensitivity analyses to increase the equivalence to the preclinical studies, including restricting analyses to individuals with minimum 3 and 7 days of BDZ exposure, and removing BDZ exposures from non-BDZ hypnotics due to the slight differences in pharmacological profiles, but these did not alter the results. The difference in the timing of BDZ exposure is perhaps most relevant to note, as MAM rats were treated peripubertally compared to in young adulthood in our study. For example, environmental enrichment—which has previously demonstrated similar effects to diazepam in preventing the psychosis phenotype in the MAM model—is not effective when given postpubertally compared to peripubertally.^39^ This is further supported by a recent neuroimaging study in FEP patients (mean age 22.7 years old) suggesting that reduced prefrontal GABA levels at baseline were associated with less functional improvement 2 years later, despite GABA levels normalizing after 2 years of antipsychotic treatment.^22^ There are also much higher levels of heterogeneity between CHR-P individuals than in MAM rats. Only approximately 25% of CHR-P individuals will transition to psychosis,^6^ and the CHR-P state is associated with a multitude of potential trajectories in terms of symptoms and functioning^8^ which may be driven by differences in pathophysiology. Preclinical studies have demonstrated that the pharmacological effect of GABAergic treatment on the subcortical dopaminergic system differs between control and MAM rats,^28^ and correspondingly BDZ effects might differ between CHR-P individuals. The heterogeneity between CHR-P individuals may also explain why no other effective preventative treatment has been discovered.^14,40^

Investigation of exploratory secondary outcomes after PSM also found no positive effects of BDZ exposure on the risk of real-world events indicative of a clinical crisis in CHR-P individuals, including psychiatric hospital admission, home visit, and A&E attendance. In fact, findings indicated a residual increased risk of receiving a home visit following BDZ exposure, albeit nonsignificant. Additionally, in our sensitivity analyses, there was a trending increased risk of subsequent A&E attendance by BDZ exposure when analysis was restricted to antipsychotic-naïve individuals, and a significant increased risk on A&E attendance when BDZ exposures from a non-BDZ hypnotic were removed from analysis. While this is the first study to examine the influence of BDZ exposure on clinical outcomes in CHR-P individuals, a recent EHR study in first-episode psychosis patients investigated the effects of antipsychotic and BDZ treatment (within the first week of illness onset) on clinical outcomes and found similar effects.^41^ BDZ treatment prior to antipsychotic treatment (vs after) increased the duration of medical and A&E admission in first-episode patients, while reducing the length of psychiatric admission.^41^ Furthermore, increased readmission to hospital has been associated with BDZ exposure in patients with chronic schizophrenia.^42,43^ An increased risk of home visit, A&E attendance, or hospitalization might reflect residual confounding-by-indication as these events capture nonspecific clinical crises influenced by a multitude of factors. For example, comorbid anxiety disorder may be associated with both BDZ exposure and repeated presentations at A&E with consequential hospital admission, creating a false association between the exposure and the event. Differences in the reason for prescription of non-BDZ hypnotics compared to traditional BDZs (sleep difficulties and anxiety/agitation, respectively) might explain why removing non-BDZ hypnotics exposed individuals from the analysis led to a significant increased risk of A&E attendance from BDZ exposure, as the analysis was restricted to individuals with a clinical profile more likely to present to A&E. Alternatively, these worse clinical outcomes could be influenced by adverse effects of BDZs including psychotic features and other adverse behavioral effects,^44^ which is important to note as in this study we do not know whether A&E attendances were related to psychiatric or other medical events. However as these effects are very rare (<1%),^45^ they are unlikely to be driving these findings in our sample.

The most common drug for BDZ exposure in this study was zopiclone/zolpidem (51%), and the most common reason for prescription of all BDZ exposures was for sleep disturbances (56%). Zopiclone/zolpidem, or “z-drugs,” are non-BDZ hypnotics with a highly similar pharmacological profile to traditional BDZs through PAM of the BDZ site on the GABA-_A_ receptor.^32^ Although CHR-P individuals do not appear to show differences in sleep architecture parameters compared to controls,^46^ reporting of sleep disturbances is very high at 55%, and is numerically higher than in individuals with early or chronic psychosis.^46^ Furthermore, sleep disturbances are associated with worse attenuated psychotic^47^ and negative symptoms,^48^ and may also contribute to transition to psychosis.^49^ Given the intricate relationship between the GABAergic system, sleep patterns, and psychosis,^50^ future studies may investigate whether the effects of BDZs on sleep disturbances contributes to their therapeutic potential in early psychosis.

This study has several strengths. We investigated the effects of BDZ exposure on transition to psychosis and clinical outcomes in CHR-P individuals, informing clinical understanding of effectiveness and safety of BDZ in this population. Secondly, we used real-world data with high ecological validity from one of the longest-established CHR-P services, affording a large sample size from a residential population with one of the highest psychosis rates worldwide.^51^ Thirdly, we used advanced statistical methods including PSM to account for confounding-by-indication, and in the process characterized disparities in BDZ exposure between CHR-P individuals that were not previously established.

This study also has some limitations. Firstly, despite using PSM to control for confounding factors, observational studies with real-world data are susceptible to confounding-by-indication. We controlled for several factors associated with BDZ exposure and adverse clinical outcomes as identified in prior research, but it is likely that there is residual confounding from additional factors that we were not statistically powered to include (eg, cannabis use, sleep disturbances). Secondly, the observational nature of the study means that factors such as treatment compliance are not known, which could impact clinical outcomes. Additionally, subsequent clinical care (eg, pharmacological/psychological interventions) beyond the exposure window but before the occurrence of events was not measured. Thirdly, as Axis I and II diagnoses are not systematically assessed as part of the OASIS baseline visit, and extraction of these data from medical records is not reliable, we were unable to examine whether the increased risk for A&E attendance in BDZ-exposed individuals was influenced by such comorbidities. Future prospective studies recording this information should expand this possibility. Fourthly, in order to maximize the follow-up time and maintain adequate power, we limited the BDZ exposure window to only 3 months after joining OASIS, as extending this window removed a significant number of individuals and transition events. Finally, limitations of the translatability of preclinical findings outlined earlier in terms of differences in BDZ compound, dose equivalence, dosing period, and timing of BDZ exposure are relevant to note.

## Conclusions

In conclusion, BDZ exposure in CHR-P individuals was not associated with a reduced risk of developing psychosis or adverse clinical outcomes after controlling for confounding-by-indication. We found suggestive evidence that prior antipsychotic exposure could be attenuating the potential therapeutic effects of BDZs in this clinical population. Further experimental research in this field is warranted, to investigate the effect of post-pubertal BDZ administration in preclinical models, investigate real-world data from child and adolescent mental health services to capture an even earlier developmental time window, and develop more selective GABAergic agents (eg, α5-GABA_A_R PAM) with better side-effect profiles and which more specifically target areas of neurobiological dysfunction in CHR-P individuals such as the hippocampus.

## Supplementary Material

Supplementary material is available at https://academic.oup.com/schizophreniabulletin/.

sbae036_suppl_Supplementary_Tables_1-2
