# Supplementary material for: Effects of Benzodiazepine Exposure on Real-World Clinical Outcomes in Individuals at Clinical High Risk for Psychosis
Source: Schizophr Bull. 2024 Apr 3;51(2):446–57. doi: 10.1093/schbul/sbae036 (PMC11908875; doi:10.1093/schbul/sbae036)
Supplement: sbae036_suppl_Supplementary_Tables_1-2 [file sbae036_suppl_supplementary_tables_1-2.docx]

**Supplementary Material**

**Effects of Benzodiazepine Exposure on Real-World Clinical Outcomes in Individuals at Clinical High-Risk for Psychosis**

Nicholas R. Livingston, MSc^1^; Andrea De Micheli, MD^2,3^; Robert McCutcheon, MD, PhD^2,4,5^; Emma Butler, MSc^2^; Marwa Hamdan, MSc^2^; Anthony A. Grace, PhD^6^; Philip McGuire, MD, PhD^4,5^; Alice Egerton, PhD^2,7^; Paolo Fusar-Poli, MD, PhD^2,3,8^; Gemma Modinos, PhD^1,9^.

**Supplementary Table 1. Details of Benzodiazepine Exposures Included in Analysis (n = 105)...........................................................................................................................page 2**

**Supplementary Table 2. Details of Medications in the Benzodiazepine-exposed Propensity Score Matched Sample (n = 105)................................................................................page 3**

**Supplementary Table 3. Additional Sensitivity Analyses in the Propensity Score Matched Sample.......................................................................................................................page 4**

**Supplementary Table 1. Details of Benzodiazepine Exposures Included in Analysis (n = 105)**

|  | | Count (%) |
| --- | --- | --- |
| BDZ name | |  |
|  | Clonazepam | 16 (15.1) |
|  | Lorazepam | 12 (11.3) |
|  | Diazepam | 16 (15.1) |
|  | Temazepam | 1 (0.9) |
|  | Zopiclone/Zolpidem | 41 (38.7) |
|  | Alprazolam | 1 (0.9) |
|  | Bromazepam | 1 (0.9) |
|  | Clonazepam + Lorazepam | 1 (0.9) |
|  | Clonazepam + Diazepam | 3 (2.8) |
|  | Clonazepam + Zopiclone | 7 (6.6) |
|  | Lorazepam + Diazepam | 1 (0.9) |
|  | Lorazepam + Zopiclone | 1 (0.9) |
|  | Diazepam + Zopiclone | 4 (3.8) |
|  | Bromazepam + Zopiclone | 1 (0.9) |
| Reason for BDZ exposure | |  |
|  | Anxiety | 17 (16.0) |
|  | Sedation | 3 (2.8) |
|  | Agitation | 13 (12.3) |
|  | Sleep | 59 (55.7) |
|  | Not known | 13 (12.3) |
|  | | **Mean (± SD)** |
| Total number of  BDZ exposure (days) | | 18.5 (25.6) |

BDZ: Benzodiazepine

**Supplementary Table 2. Details of Medications in the Benzodiazepine-exposed Propensity Score Matched Sample (n = 105)**

|  | | Count (%) |
| --- | --- | --- |
| Antipsychotic name | |  |
|  | Quetiapine | 20 (19) |
|  | Olanzapine | 19 (18) |
|  | Risperidone | 7 (7) |
|  | Aripiprazole | 6 (6) |
|  | Amisulpride | 2 (2) |
|  | Promethazine | 2 (2) |
| Antidepressant name | |  |
|  | Sertraline | 27 (26) |
|  | Citalopram | 6 (6) |
|  | Escitalopram | 2 (2) |
|  | Fluoxetine | 9 (9) |
|  | Paroxetine | 1 (1) |
|  | Mirtazapine | 5 (5) |
|  | Amitriptyline | 2 (2) |
| Other medications name | |  |
|  | Sodium Valproate | 2 (2) |
|  | Lamotrigine | 1 (1) |

**Supplementary Table 3. Additional Sensitivity Analyses in the Propensity Score Matched Sample**

A&E: Accident & Emergency; BDZ: Benzodiazepine

|  | | Transition to Psychosis | Psychiatric Hospital Admission | Home Visit | A&E Attendance |
| --- | --- | --- | --- | --- | --- |
| ≥ 3 total days of BDZ (n = 89 per group) | | | | | |
|  | HR (95% CI), *P* | 1.67 (0.89-3.11), .11 | 1.67 (0.79-3.53), .18 | 1.38 (0.86-2.23), .18 | 1.23 (0.75-2.18), .37 |
|  | Number of events;  BDZ exposed vs. unexposed | 22 vs. 18 | 16 vs. 12 | 37 vs. 31 | 33 vs. 22 |
| ≥ 7 total days of BDZ (n = 66 per group) | | | | | |
|  | HR (95% CI), *P* | 1.61 (0.75-3.45), .22 | 0.82 (0.32-2.12), .69 | 1.65 (0.94-2.89), .08 | 1.11 (0.56-2.19), .77 |
|  | Number of events;  BDZ exposed vs. unexposed | 11 vs. 7 | 7 vs. 4 | 24 vs. 12 | 16 vs. 10 |
| Removing non-benzodiazepine hypnotics (n = 75 per group) | | | | | |
|  | HR (95% CI), *P* | 0.91 (0.47-1.77), .79 | 1.41 (0.58-3.41), .44 | 1.47 (0.87-2.49), .15 | 2.08 (1.11-3.93), **.02** |
|  | Number of events;  BDZ exposed vs. unexposed | 18 vs. 17 | 13 vs. 8 | 41 vs. 28 | 31 vs. 16 |
